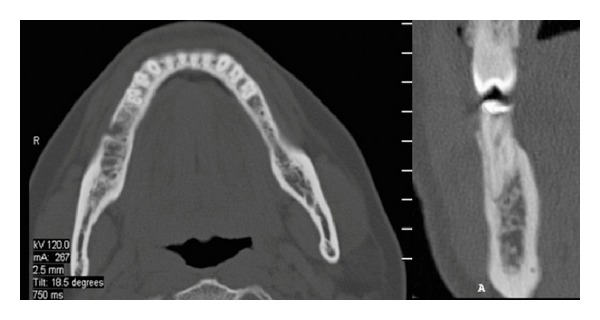# Root Canal Treatment of Mandibular Second Premolar with Three Separate Roots and Canals Using Spiral Computed Tomographic

**DOI:** 10.1155/2014/816576

**Published:** 2014-07-03

**Authors:** V. P. Hariharavel, A. Ashok Kumar, C. Ganesh, Sankar Annamalai, Kavitha Ramar, R. Aravindhan

**Affiliations:** ^1^Department of Pedodontics and Preventive Dentistry, SRM Kattankulathur Dental College and Hospital, Chennai 603203, India; ^2^Sri Ammaiappan Dental Clinic, Tamil Nadu, Chennai 600099, India; ^3^Department of Oral medicine and Radiology, SRM Kattankulathur Dental College and Hospital, Chennai 603203, India; ^4^Department of Pedodontics and Preventive Dentistry, SRM Kattankulathur Dental College and Hospital, Chennai 603203, India; ^5^Department of Oral Pathology, SRM Kattankulathur Dental College and Hospital, Chennai 603203, India

## Abstract

Anatomic and internal morphology of a root canal system is more complex and differs for each individual tooth of which mandibular premolars have earned the reputation for having aberrant anatomy. The occurrence of three canals with three separate foramina in mandibular second premolars is very rare. A wider knowledge on both clinical and radiological anatomy especially spiral computed tomographic is absolutely essential for the success of endodontic treatment. These teeth may require skillful and special root canal special shaping and obturating techniques. This paper reports an unusual case of a mandibular second premolar with atypical canal pattern that was successfully treated endodontically.

## 1. Introduction

Mandibular premolars have always been reputed to have an aberrant anatomy. Many authors have reported on these variations in the literature. Zillich and Dowson reported 11.7% occurrence of two canals and 0.4% of three canals [1] and according to Ingle, mandibular second premolars have only 12% chance of a second canal and 0.4% of a third canal. Consistently, high levels of success in endodontic treatment require an understanding of root canal anatomy and morphology. To achieve endodontic success, the entire root canal system must be debrided, disinfected, and obturated [2]. Intraoral periapical radiographs have always been considered the backbone to diagnosis but they still have their own limitations. The aim of this report was to apply a successful root canal treatment of mandibular second premolar with three separate root canals using spiral computed tomography (SCT).

## 2. Case Report

**Figure 1:**
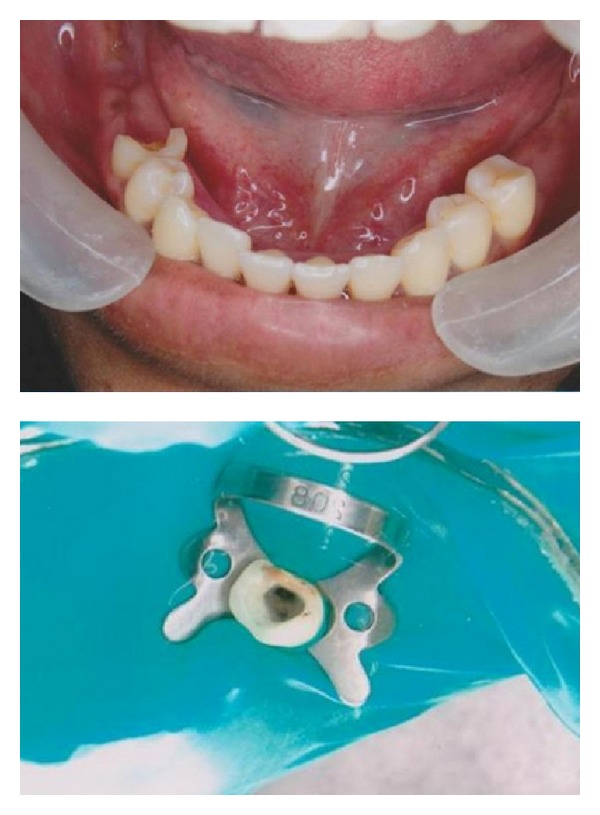


**Figure 2:**
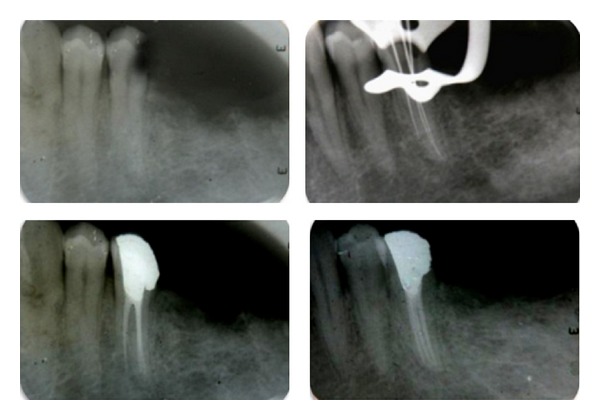


A 43-year-old female with a noncontributory medical history was referred to a clinic for endodontic treatment on the right mandibular second premolar. The chief complaint of the patient was “pain in the lower right back teeth.” Clinical examination revealed distal caries in the right mandibular second premolar tooth 45. Teeth 46, 47, and 48 were missing (Figure 1). An intraoral periapical radiograph (IOPA) was advised. Radiographic examination revealed radiolucency involving pulp with respect to tooth 45 (Figure 2). The tooth was diagnosed with irreversible pulpitis based on clinical and radiographic findings and it was decided to undergo endodontic therapy for the lower right second premolar. IOPA revealed the presence of two root canals and a third root canal was suspected due to abnormal dimension in the middle third of the root. Additional IOPA radiographs taken at different angulations could not conform the exact pathway of the third root canal as it represents only a two-dimensional image.

Informed consent was obtained from the patient for endodontic treatment of the involved teeth. The tooth was anesthetized using local anesthetic (2% Lignocaine with 1 : 100,000 epinephrine) solution by way of inferior nerve block of right side. Under rubber dam isolation, access cavity was prepared with round diamond burs in a high speed airotor hand piece. After extirpation of the pulpal tissue in the coronal part of the tooth, on entry into the pulp chamber of tooth 45 three separate canal orifices were found (Figure 1). Hence, to ascertain this rare and complex root canal anatomy of the tooth in a three-dimensional manner, dental imaging with the help of SCT was planned. Informed consent from the patient was obtained and the mandible was scanned by using SCT (Siemens Emotion 6 Slice CT scanner, SIEMENS AG, Germany). A three-dimensional reconstruction image of the mandible was obtained using DICOM CD viewer (Sienet Sky, Siemens Corporation, Germany). The involved tooth was focused and the morphology was viewed in axial sections of 0.63 mm thickness at the coronal, middle, and apical third of the roots, both mandibular first and second premolars were found to have type V (vertucci), which is very uncommon (Figure 3).

On confirming the presence of a third root canal, treatment was continued with utmost care. Gates Glidden drills 4, 3, 2 with a brushing motion were used in a crown down fashion to enlarge the orifice to obtain straight line access to all the three canals. All the three canals were negotiated with K-flex files (Dentsply Maillefer, Ballaigues, Switzerland). Working length was established with the use of K files and periapical radiographs using Ingle's method (Figure 2). Canal disinfection was performed using copious amount of 2.5% sodium hypochlorite, 17% ethylene diamine tetra acetic acid (EDTA), and saline. The canals were cleaned and shaped upto ISO 35 size master apical file with hand K files. After drying the canals with sterile paper points (Dentsply, Maillefer, Ballaiques, Switzerland), the canals were obturated by cold lateral compaction of gutta percha using zinc oxide and eugenol sealer (Dentsply, Kalsogen Plus) (Figure 2). Amalgam was used as the permanent coronal seal. Patient was advised crown for the treated tooth.

## 3. Discussion

Endodontic treatment in second mandibular premolar with a varying morphology is a challenging task. Therefore, the internal morphology must be identified precisely to achieve successful treatment. Conventional intraoral periapical radiographs are routinely employed to evaluate the root canal anatomy, but it inherently represents only a three-dimensional anatomy on a two-dimensional image [3–5]; hence, it does not allow precise assessment of complex endodontic morphology. Since its application in endodontics in 1990, CT scanning has become popular with clinicians in the past few years. Based on the results of the previous studies carried out by Kottoor et al. [6] and La et al. [7] wherein spiral CT was used for the confirmatory diagnosis of morphological aberrations in the root canal anatomy, it was opted to use CT and spiral CT in relation to tooth 45 region.

The SCT images revealed that the root canals had separate portals of exit; although the vague lines of the two roots could be observed on the periapical radiograph, the confirmatory diagnosis of the exact number of root canals could only be made with the help of SCT. These findings are clinically important as various studies have reported a failure rate of 11.45% and 4.54% of the first and second premolars. This could be due to the complex root canal anatomy of a large number of these teeth [8].

While locating the root or the canals, it should be considered in mind that the more apically the root canal divides, the more difficult it is to access and obturate efficiently. Hence, smaller K files are initially used as they can deviate buccally or lingually as the main canal divides. So a good tactile sense is important and the files have to be precurved appropriately before negotiating the canals. Also during obturation, canal patency was maintained through the apically compacted gutta purcha with a file or with a spreader of suitable taper while each canal is being obturated. Failure to recognize these extra canals and obturate them with care can lead to acute flareups during treatment and subsequent failure in endodontic therapy [8, 9] as the anatomic position of the mental foramen is in close approximity to mandibular premolars.

## 4. Conclusion

A successful nonsurgical management of mandibular second premolar with three canals has been presented. In this case extra root canal could not be confirmed with radiographs alone. Hence, the use of SCT helped us a lot in making a confirmatory diagnosis. The clinician must carefully interpret radiographs and must be vigilant on clinical inspection of the floor of the chamber for a successful treatment outcome.

## Figures and Tables

**Figure 3:**